# Immune Thrombocytopenia Coexisting With Celiac Disease and Liver Dysfunction: A Case Report of Multifactorial Complications

**DOI:** 10.7759/cureus.90503

**Published:** 2025-08-19

**Authors:** Hafiz Muhammad Faizan Mughal, Muhammad J Khan, Abdul Rehman Khalil Shaikh

**Affiliations:** 1 Internal Medicine, Khawaja Muhammad Safdar Medical College, Sialkot, PAK; 2 Acute Medicine, Midland Metropolitan University Hospital, Birmingham, GBR; 3 Pathology, Liaquat University of Medical and Health Sciences, Jamshoro, PAK

**Keywords:** autoimmune hepatitis, celiac disease, extraintestinal manifestations, gluten-free diet, immune thrombocytopenia, liver dysfunction

## Abstract

Celiac disease (CD) is a chronic disorder that influences the immune system and is also associated with abnormalities in blood and liver functions. Reports have indicated the presence of immune thrombocytopenia (ITP) and liver diseases in CD, individually, but the simultaneous occurrence of both is rare and therefore difficult to diagnose. Here, we described the case of a 49-year-old woman who had thrombocytopenia and elevated liver enzymes, but showed no gastrointestinal symptoms. However, a complete assessment led to the simultaneous diagnosis of CD, ITP, and autoimmune hepatitis. Treatment with a gluten-free diet and immunosuppressive therapy helped in both restoring the platelet counts and normalizing liver enzymes. The case illustrated that recognizing unusual symptoms in CD is important and it requires the involvement of several experts if immune comorbidities are found.

## Introduction

Celiac disease (CD) is an autoimmune condition that occurs when individuals with certain genetic markers, most commonly human leukocyte antigen DQ2 or human leukocyte antigen DQ8, consume gluten-containing products, hence triggering an immune response [1]. CD primarily damages the small intestine, but research has shown that it can cause several other symptoms that have harmful effects on the entire body. Such symptoms occur early on, before any typical gastrointestinal signs are noticed, preventing quick recognition and timely treatment [2]. Among the hematologic changes seen in CD, iron deficiency anemia, thrombocytosis, leukopenia, and, in rare cases, immune thrombocytopenia (ITP) are noticeable. ITP antibodies are the antibodies that attack the body's own platelets and cause their destruction. This autoimmune reaction is occasionally associated with CD [3].

Isolated thrombocytopenia can be the symptom of underlying CD, as seen in a few cases, but it is often misdiagnosed as a primary blood disorder instead of a symptom [4]. Liver (hepatic) involvement in CD is well documented, ranging from mild transaminase elevations ("celiac hepatitis") to severe autoimmune disorders such as autoimmune hepatitis and primary biliary cholangitis [5]. Autoimmune hepatitis is a chronic liver disorder where the immune system of the body targets liver cells, leading to inflammation, destruction of the hepatocytes, and, in severe cases, cirrhosis or fibrosis [6]. Though CD is associated with several autoimmune diseases, a simultaneous combination of ITP and liver malfunction typically arises in rare cases; therefore, accurate diagnosis becomes complicated [7]. In some cases, the disease can be misdiagnosed as an individual autoimmune disorder related to bleeding or the liver, unless a careful examination is done.

We describe the case of a woman who was 49 years old and had thrombocytopenia and elevated liver enzymes, with no gastrointestinal symptoms. Duodenal biopsies confirmed CD, and liver biopsy showed signs consistent with autoimmune hepatitis. The case demonstrated a surgical form of CD, highlighting the need to consider CD in patients with unexplained thrombocytopenia or liver dysfunction even in the absence of gastrointestinal symptoms.

## Case presentation

A 49-year-old woman was enrolled in the internal medicine clinic for a detailed checkup after reporting continued tiredness, slight bruising, and mild scleral icterus (yellowing in the eyes) that began three weeks earlier. She reported no gastric symptoms such as diarrhea, abdominal pain, bloating, or loss of appetite. The patient's records showed no history of recent infections, drinking alcohol, use of hepatotoxic drugs, or pre-existing liver disease. There were no other medical issues noted, and nobody in her family history indicated a link to autoimmune, hematologic, or gastrointestinal diseases.

During the physical assessment, the patient's skin was seen to be pale, along with a few scattered petechiae on the lower parts of her body. There was no evidence of abnormal fluid accumulation, such as ascites, pleural effusion, or pericardial effusion, in her body fluids. Her blood pressure was within normal limits, and no hepatosplenomegaly or lymphadenopathy was found. According to the record, her body mass index (BMI) was 24. Laboratory parameters at baseline and after six months of treatment are summarized in Table 1. Tests initially showed that the platelet count was markedly decreased (<150,000). Hemoglobin was slightly reduced, while the mean corpuscular volume (MCV) of red blood cells and white blood cell count were within normal limits. A peripheral blood smear showed normal morphology of white and red blood cells, but low levels of platelets. There was no sign of antibodies against blood cells in the direct antiglobulin (Coombs) test.

**Table 1:** Hematologic and biochemical parameters at presentation and after six months of treatment AST: aspartate aminotransferase; ALT: alanine aminotransferase; ALP: alkaline phosphatase; INR: international normalized ratio; MCV: mean corpuscular volume; WBC: white blood cell count; anti-tTG IgA: anti-tissue transglutaminase immunoglobulin A; anti-endomysial IgA: anti-endomysial immunoglobulin A; ANA: antinuclear antibodies; ASMA: anti-smooth muscle antibody; anti-LKM1: anti-liver-kidney microsomal antibodies; g/dL: grams per deciliter; mg/dL: milligrams per deciliter; U/L: units per liter; U/mL: units per milliliter; fL: femtoliters

Parameter	At presentation	At 6 months	Reference range
Platelet count (×10⁹/L)	36	198	150-450
Hemoglobin (g/dL)	11.8	13.9	13-17
White blood cell count (×10⁹/L)	6.3	6.1	4.0-10.0
Mean corpuscular volume (fL)	84	86	80-98
AST (U/L)	102	34	5-40
ALT (U/L)	118	29	7-56
ALP (U/L)	143	90	40-129
Total bilirubin (mg/dL)	2.1	0.8	0.2-1.2
Direct bilirubin (mg/dL)	0.9	0.2	0.0-0.3
Albumin (g/dL)	3.4	4.1	3.5-5.0
INR	1.2	1.0	0.8-1.2
Anti-tTG IgA (U/mL)	89	<10	<20
Anti-endomysial IgA	Positive (1:160)	Negative	Negative
ANA	Positive (1:160)	Negative	Negative (<1:40)
ASMA	Positive (1:80)	Negative	Negative (<1:40)
Anti-LKM1	Positive (1:40)	Negative	Negative (<1:10)

Biochemical analysis found that total bilirubin was mildly elevated (2.1 mg/dL), with the direct bilirubin of 0.9 mg/dL. The levels of serum transaminases were notably increased. The test indicated that the international normalized ratio (INR) was at the upper limit of normal and the blood albumin level was just below the normal limit. Reticulocyte count and lactate dehydrogenase (LDH) both rose a little, indicating reticulocytosis. 

A routine evaluation for autoimmune diseases and infections was performed. All the serologies for hepatitis A, B, and C, human immunodeficiency virus (HIV), and Epstein-Barr virus turned out negative. Tests for antinuclear antibodies (ANA), anti-smooth muscle antibodies (ASMA), and anti-liver-kidney microsomal antibodies (anti-LKM1) showed positive results. The ultrasound scan of the abdomen did not show any abnormality and ruled out biliary dilatation. The length of the spleen was 9.4 cm, which was within normal limits.

**Figure 1:**
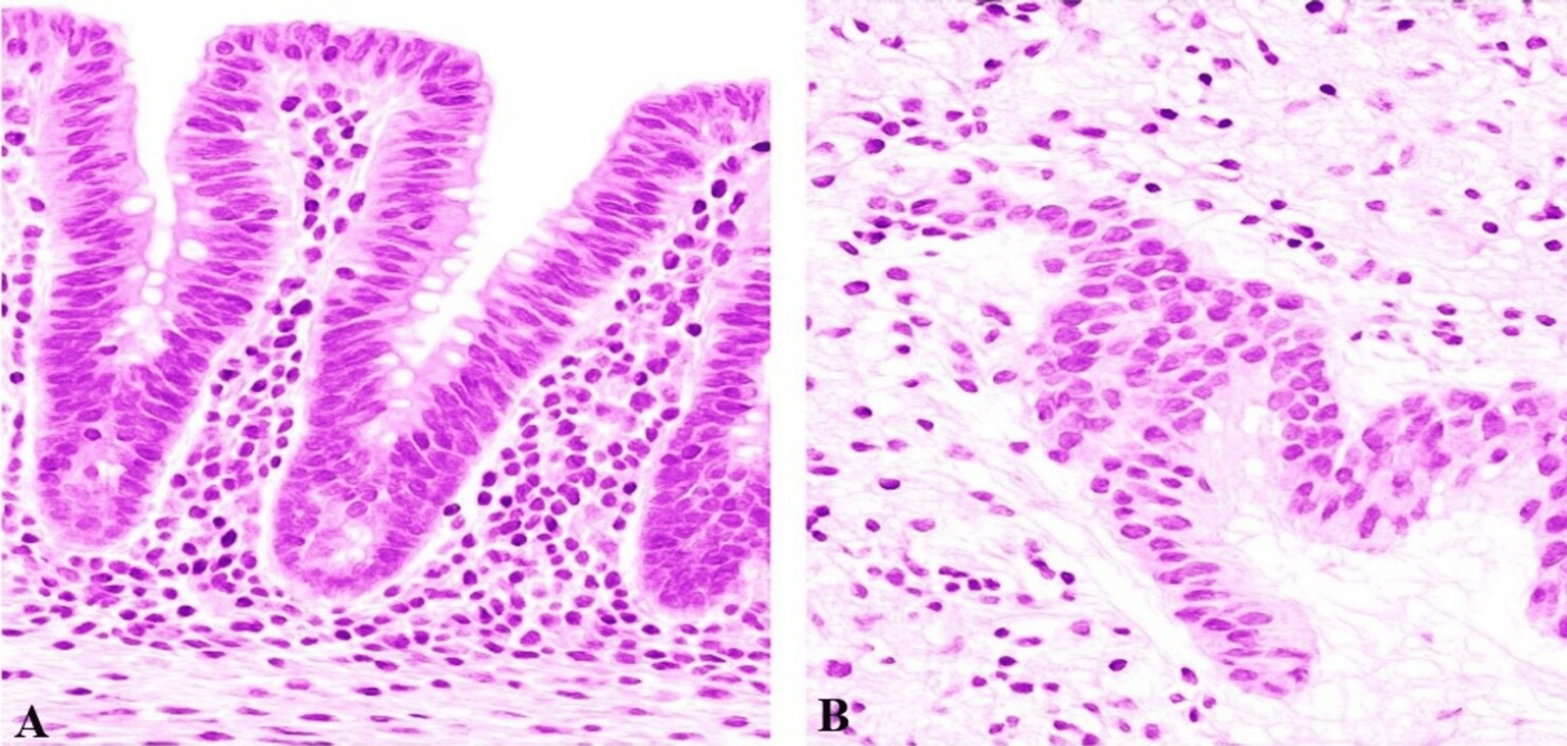
Histopathological findings from duodenum and liver biopsies (A) Duodenal biopsy showing villous blunting, crypt hyperplasia, and increased intraepithelial lymphocytes consistent with Marsh classification stage IIIa (H&E stain, 20×). (B) Liver biopsy showing interface hepatitis with lymphoplasmacytic infiltrate and mild portal fibrosis, characteristic of autoimmune hepatitis (H&E stain, 40×).

Due to unexplained low platelets and rising liver enzymes, it was decided to run more specific immunological tests. Based upon this information, an upper gastrointestinal endoscopy was ordered. The duodenal mucosa had no apparent abnormalities during endoscopy, but biopsies taken from the second part of the duodenum showed that some parts of the villi were atrophied, crypts were hyperplastic, and there were excess lymphocytes in the epithelium, indicating Marsh classification stage IIIa (A). To determine the cause of her liver problems, a biopsy of the liver was carried out at the same time. Histological examination found inflammation at the hepatocyte surface, with lymphocyte and plasma cell infiltration, along with very slight fibrosis in the portal tract, which are signs of autoimmune hepatitis (B) as shown in Figure 1.

The liver tissue did not have signs of steatosis, cholestasis, or viral inclusions. A summary of the findings from biopsies of the duodenum and liver is given in Table 2.

**Table 2:** Duodenal and hepatic histopathology findings IEL: intraepithelial lymphocytes

Biopsy site	Findings	Interpretation
Duodenum (initial)	Villous blunting, crypt hyperplasia, IEL >40/100 cells	Marsh IIIa: active celiac disease
Duodenum (12 months)	Normal villous architecture, IEL <25/100 cells	Mucosal recovery with gluten-free diet
Liver	Interface hepatitis, lymphoplasmacytic infiltrate, mild portal fibrosis	Autoimmune hepatitis I

**Figure 2:**
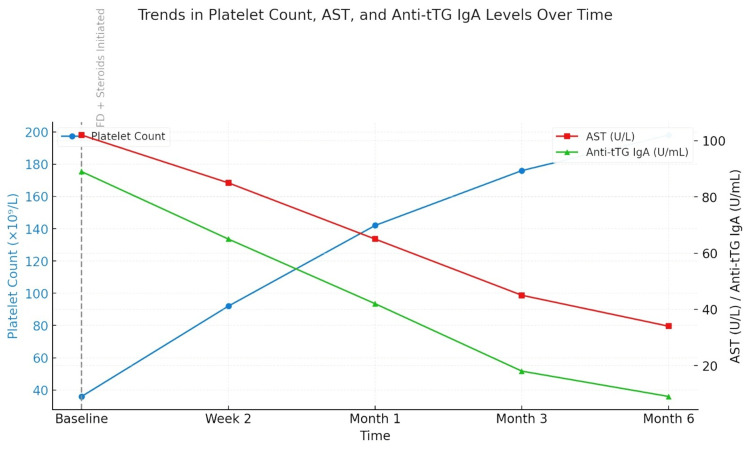
Trends in platelet count, AST, and anti-tTG IgA levels over time AST: aspartate aminotransferase; anti-tTG IgA: anti-tissue transglutaminase immunoglobulin A

During her initial diagnosis of CD, the patient was found to have ITP and autoimmune hepatitis. She received a gluten-free diet and was given prednisolone tablets at a dose of 1 mg for every kilogram of her mass daily which was gradually cut down over the next eight weeks. There was a quick response to treatment; three weeks after starting, her platelet count reached 142×10⁹/L, and her serum transaminases began to return to normal levels. After two months, both the blood and liver tests were normal, and the patient said that the fatigue was gone. As seen in Figure 2, the changes in platelet count, aspartate aminotransferase (AST), and anti-tissue transglutaminase immunoglobulin A (anti-tTG IgA) with time show signs of improvement.

At six months, anti-tTG IgA levels went below 10 U/mL, which demonstrated the patient was consistently on the gluten-free diet. Twelve months later, a new duodenal biopsy showed normal villi and a 3:2 ratio of villi to crypts, and only a few intraepithelial lymphocytes were seen. At the checkup after one year, the patient still showed no signs of disease and normal lab results and was sticking to her diet. Thrombocytopenia and hepatic dysfunction did not happen again.

## Discussion

CD is an autoimmune condition with a variety of symptoms that can appear all over the body and not just in the intestines [8]. While iron-deficient anemia is the most common symptom, CD is also associated with an increased risk of immune-mediated hematological disorders and liver complications [9]. Clinically, the occurrence of both thrombocytopenia and autoimmune liver disorder in CD is rare and often overlooked. In this case report, a middle-aged woman experienced thrombocytopenia and transaminitis without any gastrointestinal problems. The results of further serology and biopsy established a diagnosis of CD along with changes similar to those of autoimmune hepatitis. The presence of these three specific problems together, i.e., ITP, liver dysfunction, and CD, caused a distinct complication that called for not only a wide range of diagnostics but also an organized approach to treatment.

Reportedly, these findings commonly follow the childhood or general autoimmunity trend; however, in our case, adult-onset isolated ITP indicated an association with CD [10]. The patient was also dealing with problems from autoimmune liver disease. In a study by Olén et al., the Swedish patients with biopsy-verified CD were at nearly twice the risk of ITP (HR 1.91) and nearly three times the risk of chronic ITP (HR 2.77) [11]. It is seen to be common for people with CD to develop increased liver enzymes, referred to as celiac hepatitis, which typically gets better after following a gluten-free diet [12]. Our patient's liver tissues revealed interface hepatitis with lymphoplasmacytic changes that were typical of autoimmune hepatitis. And since anti-LKM1, ANA, and ASMA were positive, this added to the evidence that the liver inflammation was autoimmune. A number of studies suggest that untreated CD allows intestinal contents to leak, leading to immune activation in other parts of the body and risk of hepatic autoimmunity. This would suggest that hepatic dysfunction could be more than a simple side effect; it could be a systemic response by the immune system, set off by gluten intake [13].

When classical symptoms are not present, diagnosing the disease can be slow, which can lead to light or delayed treatment of reversible autoimmune diseases. However, when a patient shows improvement on a gluten-free diet and corticosteroid therapy, then it strongly indicates the cause to be immune-related. After a few months, in this case patient, liver enzymes, platelets, and celiac blood markers returned to normal, and the repeat duodenal biopsy showed no signs of inflammation. It supports the theory that restricting dietary antigens and modifying the immune system with the help of a gluten-free diet can prevent adverse effects caused by CD.

The report explains why doctors should suspect CD when patients have abnormal platelet counts or liver function, even if they don't have gastrointestinal symptoms. Collaboration among professionals is necessary to prevent poor diagnosis and to take action promptly. Further study is needed to learn more about the causes and future outcomes of this multifactorial form of CD.

## Conclusions

This case highlights the systemic nature of CD, which is often underdiagnosed due to its presentation without classical gastrointestinal symptoms, but still leads to severe immune-mediated complications, including ITP and autoimmune hepatitis. Coexistence of both hematological and hepatic disruptions in a recently diagnosed seropositive CD patient highlights the possible pathophysiological connection between gluten sensitivity and the wider autoimmune malfunction.

The clinically proven effect of a strictly gluten-free diet in combination with immunosuppressive therapy additionally confirms the importance of gluten as a triggering agent of immune reaction. These results underline the need to account for the possibility of CD in patients with unexplained hematologic or hepatic abnormalities, even without digestive symptoms, in order to intervene on time and to avoid additional immune-mediated damage.
